# Telemedicine Bridge Between EDs and NHs: Reducing NH Residents’ Avoidable Emergency Visits

**DOI:** 10.1093/geroni/igaf122.695

**Published:** 2025-12-31

**Authors:** Hanzhang Xu, Chong Yau Ong, Angus Jun Jie Ng, Jiameng Yuan, Gayathri Devi Nadarajan, Marcus Ong, Jean Mui Hua Lee

**Affiliations:** Duke University, Durham, North Carolina, United States; Sengkang General Hospital, Singapore, Singapore; Sengkang General Hospital, Singapore, Singapore; Duke University, Durham, North Carolina, United States; Singapore General Hospital, Singapore, Singapore; Duke-NUS Medical School, Singapore, Singapore; Sengkang General Hospital, Singapore, Singapore

## Abstract

Nursing Home (NH) residents have high rates of emergency department (ED) visits, with nearly half potentially avoidable. We evaluated the impact of the EAGLEcareACT (Enhancing Advance Care Planning, Geriatrics, and End-of-Life Care Acute Care Team) --an interdisciplinary telemedicine program at Sengkang General Hospital (SKH), Singapore linking ED physicians with NH staff—on reducing avoidable ED visits among NHs residents. Between 2020-2023, 7 out of the 35 NHs in the catchment area were enrolled in EAGLEcareACT. We analyzed data from all ED visits at SKH from NHs residents 2020-2023. Outcomes included hospitalization, National Early Warning Score (NEWS), and ED acuity levels. We used generalized estimating equations models, to assess associations between EAGLEcareACT enrollment and outcomes accounting for within-NH clustering. Among 6,344 ED visits from NH residents (median age 78 years, 37.8% female, 85.8% Chinese), 2,483 (39.1%) were from EAGLEcareACT partnered NHs. Patients from partnered NHs were slightly older (median 79 vs. 78 years) and more likely to be female (43.4% vs. 34.2%) than patients from non-partnered NHs. After adjustment, comparing to patients from non-partners NHs, those from partnered NHs were more likely to be hospitalized (odds ratio [OR] 1.59, 95% CI 1.10-2.29), have a critical condition as NEWS scores ≥5 (OR 1.76, 95% CI 1.27-2.45), and present with high acuity (OR 1.69, 95% CI 1.15-2.50). These findings suggest that EAGLEcareACT effectively manages non-urgent cases at NHs via telemedicine while appropriately identifying high-risk older adults requiring ED care. Further research is needed to evaluate long-term outcomes and healthcare utilization impacts.

